# Is It Possible to Train the Focus on Positive and Negative Parts of One’s Own Body? A Pilot Randomized Controlled Study on Attentional Bias Modification Training

**DOI:** 10.3389/fpsyg.2019.02890

**Published:** 2019-12-20

**Authors:** Nicole Engel, Manuel Waldorf, Andrea Hartmann, Anna Voßbeck-Elsebusch, Silja Vocks

**Affiliations:** ^1^Department of Clinical Psychology and Psychotherapy, University of Osnabrück, Osnabrück, Germany; ^2^Department of Clinical Psychology and Psychotherapy, University of Münster, Münster, Germany

**Keywords:** attentional bias modification training, dot-probe task, body dissatisfaction, attentional bias, body image

## Abstract

Dysfunctional body- and shape-related attentional biases are involved in the etiology and maintenance of eating disorders (ED). Various studies suggest that women, particularly those with ED diagnoses, focus on negatively evaluated parts of their own body, which leads to an increase in body dissatisfaction. The present study aims to empirically test the hypothesis that non-ED women show an attentional bias toward negative body parts and that the focus on positive and negative parts of one’s own body can be modified by attentional bias modification training based on a dot-probe task. Although several studies have measured body-related attentional biases by using pictures of participants’ own bodies, the approach of investigating attentional bias *via* a dot-probe task while presenting pictures of the participants’ own body parts and modifying the biased attention using such pictures is novel. Women (*n* = 60) rank-ordered 10 parts of their own body regarding their attractiveness. To examine and modify the attentional focus, pictures of the self-defined positive and negative parts of one’s own body were presented by means of a dot-probe task. A paired-sample *t*-test revealed no difference between reaction times to negative compared to positive body parts, indicating no attentional bias toward negative parts of one’s own body. A two-way ANOVA revealed a main effect of time for pictures of positive and negative parts of one’s own body, with a decrease in reaction times from pre- to post-training. However, there was no significant interaction between time and training condition concerning reaction times to positive and negative body parts. Our findings replicate previous evidence of a balanced attentional pattern regarding one’s own body in women without ED diagnoses. However, the dot-probe task failed to modify the attentional focus. As the modifiability of state body image increases with more pronounced body dissatisfaction, the next step would be to test this approach in clinical samples of women with ED diagnoses.

## Introduction

Body image disturbance is a core feature of anorexia and bulimia nervosa according to the Diagnostic and Statistical Manual of Mental Disorders, fifth edition (DSM-5; American Psychiatric Association, 2013) and plays a significant role in the etiology and maintenance of eating disorders (Westerberg-Jacobson et al., 2010; Stice et al., 2011; Kearney-Cooke and Tieger, 2015). The integrated cognitive-behavioral model of eating disorders (Williamson et al., 2004) posits that overconcern with shape and weight leads to a body- and eating-related negative self-schema, which is activated by body- or food-related cues. The activation of the self-schema, in turn, leads to attentional biases toward negatively evaluated body- and food-related stimuli. These biases give rise to negative cognitive-affective states such as body-related anxiety and dissatisfaction, which, according to the theory, trigger body checking behavior, body avoidance, or purging behavior.

Various studies have confirmed the model’s assumption that women with eating disorder symptoms show an attentional bias toward negatively evaluated body- and shape-related stimuli (for a review, see Aspen et al., 2013; Schuck et al., 2018). Within this field of research, the dot-probe task (MacLeod et al., 1986; MacLeod, 2012) is a prominently applied paradigm for investigating attentional biases in eating disorders. In the dot-probe task, two competing disorder-relevant stimuli, i.e., words or pictures with different emotional valence are simultaneously presented on a screen. Subsequently, a target probe is presented at the location of one of the stimuli, to which the participant is prompted to react. Shorter reaction times to the target indicate that the participant’s attention was directed toward the target location just before the target appeared. Hence, reaction time is a measure for the participant’s attentional focus and provides information about disorder-specific attentional processes (MacLeod et al., 1986).

The clinical relevance of attentional biases toward negative body- and shape-related stimuli has been repeatedly examined (for a review, see Kerr-Gaffney et al., 2019). In their study using the dot-probe task, Gao et al. (2011) found that women with a high degree of body dissatisfaction showed a faster and prolonged visual attention toward weight-associated words compared to women with a low degree of body dissatisfaction. Furthermore, Rieger et al. (1998) reported that patients with anorexia and bulimia nervosa directed their attention toward negatively evaluated body- and shape-related words, as they showed shorter reaction times to theses stimuli compared to neutral and positive stimulus words in a dot-probe task. In accordance with this finding, Shafran et al. (2007) found that women with eating disorder diagnoses showed an attentional bias toward negative eating- and shape-related pictures in a dot-probe task compared to participants in the control group.

In addition to these studies investigating the attentional bias toward negative body- and shape-related stimuli in general, there are several findings that women with eating disorder diagnoses display a biased attention toward their own body. For example, in an experimental analysis using a dot-probe task with pictures of participants’ own bodies and pictures of other people’s bodies, Blechert et al. (2010) detected shorter reaction times to pictures of one’s own body in women with anorexia nervosa diagnoses. Moreover, in an eye-tracking study using pictures of participants’ own bodies, Bauer et al. (2017) found that women with anorexia and bulimia nervosa diagnoses displayed an attentional bias toward parts of their own body which were self-evaluated as unattractive. This finding is in line with the results of an eye-tracking study by Tuschen-Caffier et al. (2015), who also showed that women with anorexia and bulimia nervosa diagnoses focused more on subjectively unattractive parts of their own body than on subjectively beautiful body parts. Non-clinical participants, in contrast, showed a more balanced attentional pattern concerning different parts of their own body. In this regard, Jansen et al. (2005) even reported that women without eating disorder diagnoses displayed a self-serving attentional focus regarding their own body, by directing their attention toward subjectively positive body parts rather than toward subjectively negative body parts.

However, contrary to the latter finding, several studies suggest that women without eating disorder diagnoses also display an attentional bias toward negatively valenced parts of their own body. For example, in an eye-tracking study, Roefs et al. (2008) found that non-clinical women displayed an attentional focus on self-defined unattractive body parts. Moreover, Bauer et al. (2017) reported that non-clinical adolescent females showed an attentional bias toward subjectively unattractive parts of their own body. These inconsistent findings might be explained by the assumption of the integrated cognitive-behavioral model of eating disorders (Williamson et al., 2004) that the extent of biased attention is linked to the degree of body dissatisfaction, leading to a more pronounced attentional bias in women with high levels of body dissatisfaction (Roefs et al., 2008; Bauer et al., 2017) compared to women with low levels of body dissatisfaction (Tuschen-Caffier et al., 2015).

Although, as described above, several studies have examined body-related attentional biases using the dot-probe paradigm, there is no research investigating attentional bias toward negatively evaluated parts of one’s own body by means of a dot-probe task and employing pictures of the participants’ own body parts. Such an approach is important, as the use of pictures of the participants’ own body parts might enhance the ecological validity of findings.

As predicted by the integrated cognitive-behavioral model of eating disorders (Williamson et al., 2004), dysfunctional body- and shape-related attentional biases seem to be involved in the development and maintenance of eating disorders (Cash, 2011; Smeets et al., 2011b). Several correlational studies suggest that an attentional bias toward negatively evaluated body-related stimuli leads to an increase in symptoms associated with eating disorders, such as body dissatisfaction, body checking, and body avoidance behavior (for a review, see Lee and Shafran, 2004).

To overcome the limitations of correlational research and to allow for causal inferences, several studies have attempted to examine the effects of attentional bias modification training (ABMT) on eating disorder-related symptoms. In order to induce an attentional bias by means of a modified dot-probe task, in every trial, the target probe appears exclusively at the location of the stimuli to which the participant is trained to attend (MacLeod et al., 2002). Accordingly, to induce an attentional bias toward positive stimuli, the target probe appears exclusively at the location of the positive stimuli, whereas to induce an attentional bias toward negative stimuli, the target probe appears exclusively at the location of the negative stimuli. For example, in a study by Smith and Rieger (2009), non-clinical women were trained to focus on negative shape- and weight-related words in the framework of a modified dot-probe task. The authors found that ABMT toward negative shape- and weight-related words exacerbated body dissatisfaction, while ABMT toward positive or neutral shape- and weight-related words did not. Similarly, in an eye-tracking study, Smeets et al. (2011a) induced an attentional bias toward self-defined attractive and unattractive body parts. Again, after the ABMT on unattractive body parts, a decrease in body satisfaction was observed.

Taken together, the results of these experimental studies indicate that an attentional bias might contribute to the development and maintenance of body dissatisfaction, which is regarded as a manifestation of body image disturbance (Hrabosky et al., 2009). Therefore, ABMT might be a promising interventional approach for increasing body satisfaction and for the treatment of body image disturbance in women with eating disorders.

While in other areas such as anxiety disorder research, there is an abundance of studies on ABMT, albeit with heterogeneous findings (for a review, see Bar-Haim, 2010; Hakamata et al., 2010), only a small number of studies have attempted to modify the attentional bias toward eating disorder-relevant stimuli (Smith and Rieger, 2006, 2009; Werthmann et al., 2014). Moreover, only one experimental study has investigated the effects of ABMT using pictures of participants’ own body parts (Smeets et al., 2011a). In this eye-tracking study, non-clinical women were trained to focus on either self-defined attractive or unattractive parts of their own body. For this purpose, a probe was presented on attractive or unattractive body parts of a blurred picture of the participants’ body. After detecting the probe, which was measured by an eye tracker, the corresponding body part lit up until the next trial. After the ABMT on subjectively unattractive body parts, self-reported body satisfaction decreased. Correspondingly, subsequent ABMT on attractive body parts led to an increase in body satisfaction. This finding indicates that ABMT might be a promising approach for modulating dysfunctional attentional processes. However, as this study did not use a standard dot-probe paradigm, it remains unclear how its outcome compares to results from this line of ABMT research. Moreover, it can be argued that the presentation of competing body stimuli might increase the ecological validity and generalizability of findings. The simultaneous presentation of competing body parts in the dot-probe task might reflect the participants’ experience in daily life, as participants have the opportunity to focus on different parts of their own body.

To sum up, several studies have examined the attentional bias concerning body-related words (Rieger et al., 1998; Gao et al., 2011) or pictures (Shafran et al., 2007; Blechert et al., 2010) using the dot-probe paradigm, but no study has investigated attentional bias toward parts of one’s own body by means of a dot-probe task and employing pictures of participants’ own body parts. To date, attentional bias toward specific parts of one’s own body has been assessed in various eye-tracking studies (Jansen et al., 2005; Roefs et al., 2008; Smeets et al., 2011a; Tuschen-Caffier et al., 2015; Bauer et al., 2017). Moreover, only a small number of studies have attempted to examine the modification of attentional bias *via* ABMT based on a dot-probe task, and these studies used body-related words (Smith and Rieger, 2006, 2009) or food-related pictures (Werthmann et al., 2014) as stimuli. To date, no study investigated the modification of attentional bias toward parts of one’s own body by means of a modified dot-probe task and employing pictures of participants’ own body parts. Besides, only one eye-tracking study (Smeets et al., 2011a) intended to modify the attentional focus on parts of participants’ own body. In addition, no studies have attempted to examine the attentional bias and modify the attentional focus within the framework of a single study. Hence, no previous study has investigated the attentional bias toward negatively valenced parts of one’s own body while also examining ABMT by means of a dot-probe task including pictures of participants’ own body parts. Therefore, the aim of the present study was to fill this gap by examining whether non-clinical women display an attentional bias toward negatively evaluated parts of their own body within the framework of a dot-probe task. Furthermore, we wished to test whether attentional bias can be modified by ABMT based on a dot-probe task comprising pictures of the participants’ own body parts ranked according to different levels of attractiveness. Finally, we sought to investigate whether the degree of body satisfaction changes from pre- to post-ABMT.

For this purpose, 60 non-clinical females underwent the ABMT with 20 participants allocated to each of the following three different training conditions: positive, negative, and control. The positive training condition aimed to induce an attentional bias toward positive parts of one’s own body, whereas the negative training condition aimed to induce an attentional bias toward negative parts of one’s own body. The control condition did not aim to modify participants’ attention. To determine the participants’ attentional focus, reaction times to the target probe were measured.

First, we hypothesized that non-clinical women would display an attentional bias toward negatively valenced parts of their own body within the framework of a dot-probe task, as indicated by shorter reaction times to pictures of negatively evaluated body parts compared to reaction times to pictures of positively evaluated body parts. Second, we expected that following ABMT on positively evaluated parts of one’s own body, reaction times to pictures of positively evaluated body parts would be shorter compared to ABMT on negatively evaluated body parts and the control condition. Third, we hypothesized that following ABMT on negatively evaluated parts of one’s own body, reaction times to pictures of negatively evaluated body parts would be shorter compared to ABMT on positively evaluated body parts and the control condition. Fourth, we expected that the degree of body satisfaction would increase from pre- to post-ABMT in the case of ABMT on positively evaluated parts of one’s own body, decrease in the case of ABMT on negatively evaluated parts of one’s own body, and remain unchanged in the control condition. Fifth, we hypothesized that the more pronounced the attentional bias before the ABMT, the higher the change in the degree of body dissatisfaction from pre- to post-ABMT.

## Materials and Methods

### Participants

The sample consisted of *N* = 60 female students from the University of Osnabrück, Germany, who received course credits for their participation in the current study. Inclusion criteria were female sex and age between 18 and 40 years. Exclusion criteria were a current pregnancy, conspicuous tattoos, body piercings, birthmarks or port-wine stains, physical deformities, and large skin wounds. These exclusion criteria were selected in order to avoid an attention allocation toward conspicuous bodily characteristics. Moreover, certain characteristics (e.g., scars) might influence the emotional valence of body parts. In a related vein, these characteristics further reduce the similarity of body stimuli and thus threaten internal validity. The exclusion criterion of a current pregnancy was selected for ethical reasons. The study was approved by the ethics committee of the University of Osnabrück.

### Assessment of Eating and Body Image Pathology

The Eating Disorder Examination-Questionnaire (EDE-Q; Fairburn and Beglin, 1994; German-language version: Hilbert et al., 2007) assesses the extent of eating disorder symptoms in the past 28 days. The self-report questionnaire consists of 27 items generating the four subscales “Restraint,” “Eating concern,” “Weight concern,” and “Shape concern.” Each item is rated on a seven-point Likert-type scale ranging from 0 (= “no days”/“not at all”) to 6 (= “every day”/“markedly”). The internal consistency of the different subscales of the German-language version ranges from *α* = 0.85 to *α* = 0.93. The test-retest reliability lies between *r*_tt_ = 0.67 and *r*_tt_ = 0.85 (Hilbert et al., 2007).

The Body Checking Questionnaire (BCQ; Reas et al., 2002; German-language version: Vocks et al., 2008b) is a self-report questionnaire assessing the frequency of specific body-related checking behavior. It consists of 23 items, which are rated on a five-point Likert-type scale ranging from 0 (= “never”) to 4 (= “very often”). For the German-language version, the internal consistency is *α* = 0.93 for females with eating disorder pathology and *α* = 0.90 for non-clinical females (Vocks et al., 2008b). The test-retest reliability for a sample of non-clinical females is *r*_tt_ = 0.88 (Vocks et al., 2008b).

The Body Image Avoidance Questionnaire (BIAQ; Rosen et al., 1991; German-language version: Legenbauer et al., 2007) is a self-report questionnaire consisting of 19 items which assess the frequency of body-related avoidance behavior and behavioral tendencies which are associated with body-image disturbances on the four subscales “Clothing,” “Social activities,” “Restraint,” and “Grooming and weighing.” Each item is rated on a five-point Likert-type scale ranging from 0 (= “never”) to 4 (= “always”). The internal consistency of the different subscales of the German-language version ranges from *α* = 0.64 to *α* = 0.80. The test-retest reliability lies between *r*_tt_ = 0.64 and *r*_tt_ = 0.81 (Legenbauer et al., 2007).

The Body Image States Scale (BISS; Cash et al., 2002) is a six-item self-report questionnaire assessing state body satisfaction. The items refer to physical appearance, shape, weight, physical attractiveness, and comparison with one’s usual body satisfaction as well as with an average person’s appearance. Each item is answered on a nine-point Likert-type scale ranging from 0 (= “dissatisfied/unattractive”) to 8 (= “satisfied/attractive”). The internal consistency ranges from *α* = 0.77 to *α* = 0.90, and the test-retest reliability is *r*_tt_ = 0.69 (Cash et al., 2002).

### Stimulus Material

To modify the participants’ attentional focus *via* the dot-probe task (MacLeod et al., 1986), pictures of the participants’ positively and negatively evaluated body parts were created.

In order to create these pictures, participants initially rated 10 parts of their own body, i.e., belly, breast, buttocks, lower legs/feet, thighs, upper back, lower back, upper arms, forearms/hands, and décolleté, concerning their attractiveness, with “1” the least attractive and “10” the most attractive body part. Subsequently, for each participant, four full-body pictures were taken in front of a plain white background, with participants wearing standardized gray underwear and in four different standardized poses, i.e., front view with arms bent, back view with arms bent, front view with arms stretched upwards, and lateral view with arms stretched forwards. The full-body pictures were then cut into image sections of the 10 different body parts using an image editing program. Body part pictures had a dimension of 200 × 120 pixels. Finally, each body part picture was embedded in the dot-probe task in order to deploy them as positive and negative stimuli during the ABMT. The participants’ five most attractively rated body parts were used as positive stimuli and the five least attractively rated were used as negative stimuli in the attentional training.

### Attentional Bias Modification Training

**Figure 1:**
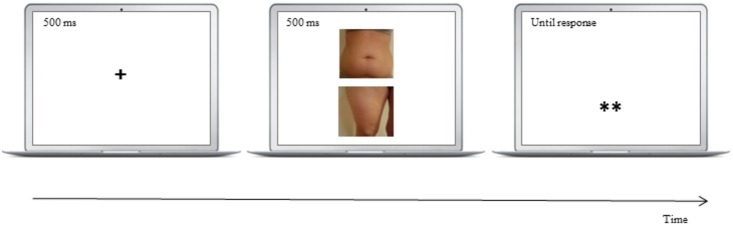
Sequence of a dot-probe task trial showing an example of stimuli used in the dot-probe task. Note: ^+^fixation cross; **target probe.

In order to examine and modify the participants’ attention concerning specific parts of their own body, the dot-probe task according to MacLeod et al. (1986, 2002) was conducted. This version of the task has been applied in diverse previous studies examining attentional bias and its modification in different research fields (Shafran et al., 2007; Smith and Rieger, 2009; Bar-Haim, 2010; Hakamata et al., 2010) and has led to important findings on attentional bias toward disorder-specific stimuli. We employed attentional training *via* the dot-probe task, with three different training conditions comprising a positive, negative, and control condition (MacLeod et al., 1986; Renwick et al., 2013). Participants were seated at about 60 cm viewing distance in front of a monitor. No chin rest was used. At the beginning of the dot-probe task, a fixation cross was presented in the center of the screen for 500 ms (see Figure 1). Subsequently, two competing pictures of a positive and a negative part of the participants’ body appeared for 500 ms, with one picture presented on the upper half and one on the lower half of the screen with a distance of 3 cm. Following the presentation of the pictures, a target probe, either “*” or “**,” was shown at the location of the positive or the negative picture. The location of the target probe depended on the training condition: In the positive training condition, for inducing an attentional bias toward positive parts of one’s own body, the target probe appeared exclusively at the location of the positive pictures. In the negative training condition, for inducing an attentional bias toward negative parts of one’s own body, the target probe appeared exclusively at the location of the negative pictures. In the control condition, the target probe appeared randomly, with equal frequency, at either location. Participants were instructed to react to the target probe as quickly and accurately as possible by pressing the “1”″ key on the keyboard for “*” and the “2”″ key for “**.”

### Hardware and Software

For presenting the dot-probe task, a 17″ TFT LCD Iiyama monitor with a 1,280 × 1,024 pixel definition and the E-Prime-software (version 2.0) were used. The participants’ full-body pictures were taken using a Nixon Coolpix L120 automatic camera on a tripod. To generate the image sections showing the participants’ body parts, the picture editing software GIMP 2 was used.

### Procedure

First, participants were informed about the course of the study and written consent was obtained from each participant without disclosing the aim of the investigation at that time. As a cover story, participants were told that the study was an assessment of reaction times. Participants were informed that during the task, the pictures of their own body parts would be presented on a screen. After generating the pictures of the body parts, which served as positive and negative stimuli in the ABMT, participants completed the BISS and were asked to carry out the dot-probe task presented on a screen. First, participants underwent three test trials using pictures of furniture as neutral stimuli in order to rectify any individual problems concerning the dot-probe task. Second, the pre-training measurement was administered. In 100 trials, the target probe randomly occurred with equal frequency at the location of pictures of positive and negative parts of one’s own body. Third, the ABMT was carried out. For this purpose, participants were randomly allocated to the positive, the negative, or the control condition of the dot-probe task, each comprising 500 training trials. This was followed by 100 trials of the post-training measurement, which exactly mirrored the pre-training measurement. After the ABMT, participants again completed the BISS and subsequently the self-report questionnaire battery.

### Statistical Analyses

Data analysis was conducted using the Statistical Package for the Social Sciences (SPSS, Version 25.0). First, the three training groups were compared in terms of age, body mass index, and their scores on the EDE-Q, BCQ, and BIAQ using a one-way ANOVA including Levene’s test of homogeneity of variances.

In order to compare the reaction times to pictures of positive and negative parts of one’s own body, a paired-sample *t*-test was conducted.

Furthermore, in order to investigate the reaction times to pictures of positive and negative parts of one’s own body before and after the ABMT, a two-way ANOVA with the within-subjects factor Time (i.e., reaction times before and after the ABMT) and the between-subjects factor Group (i.e., positive, negative, and neutral training condition) was conducted.

Additionally, in order to examine the BISS scores before and after the ABMT, a two-way ANOVA with the within-subjects factor Time (i.e., before and after the ABMT) and the between-subjects factor Group (i.e., positive, negative and neutral training condition) was conducted.

In order to examine the association between attentional bias before the ABMT and the change in the degree of body dissatisfaction from pre- to post-ABMT, product-moment correlation coefficients were computed, with respect to the positive condition and the negative condition of the ABMT. To represent the extent of attentional bias before the ABMT, we calculated a bias score by subtracting reaction times to negatively evaluated body parts from reaction times to positively evaluated body parts. To represent the change in the degree of body dissatisfaction from pre- to post-ABMT, the degree of body dissatisfaction after the ABMT was subtracted from the degree of body dissatisfaction before the ABMT.

For all analyses, the significance level was set at *p* < 0.05 (two-tailed).

## Results

### Participants’ Characteristics

Participants’ characteristics are presented in Table 1. No significant group differences between participants in the positive training condition, the negative training condition, and in the control condition were found for age, body mass index, and the questionnaire measures.

**Table 1:** Means (*M*) and standard deviations (SD) of participants’ characteristics and questionnaire measures.

Dependent variable	Total sample		Positive condition		Negative condition		Control condition		Group comparison		
	*M*	SD	*M*	SD	*M*	SD	*M*	SD	*F*	df	*p*
Age	22.49	2.94	23.25	3.51	21.85	2.74	22.37	2.41	1.17	2	0.319
BMI	21.81	2.48	22.37	3.08	22.01	2.07	21.07	2.09	1.48	2	0.236
EDE-Q	1.01	0.76	0.96	0.82	1.13	0.74	0.93	0.75	0.38	2	0.684
BCQ	0.68	0.34	0.68	0.38	0.59	0.18	0.78	0.42	0.81	2	0.453
BIAQ	0.42	0.43	0.47	0.51	0.38	0.27	0.40	0.47	0.25	2	0.782

*Age in years; BMI, body mass index in kg/m^2^; EDE-Q, Eating Disorder Examination-Questionnaire; BCQ, Body Checking Questionnaire; BIAQ, Body Image Avoidance Questionnaire; F, F value; df, degrees of freedom; p, p value*.

There were no differences in reaction times to positive parts of one’s own body between the three groups before the ABMT was executed, *F*(2, 57) = 0.07, *p* = 0.936. Moreover, there were no differences in reaction times to negative parts of one’s own body between the three groups before the ABMT was executed, *F*(2, 57) = 0.04, *p* = 0.965.

### Comparison of the Reaction Times (in Milliseconds) to Pictures of Positive and Negative Parts of One’s Own Body Before the Attentional Bias Modification Training

The results of a paired-sample *t*-test revealed no differences in reaction times to negative parts of one’s own body compared to positive parts of one’s own body in the context of the dot-probe task before the ABMT was executed, *t*(59) = 1.16, *p* = 0.251, *d* = 0.04. This finding indicates that there is no attentional bias toward negative parts of one’s own body in non-clinical women.

### Comparison of the Reaction Times (in Milliseconds) to Pictures of Positive and Negative Parts of One’s Own Body Before and After the Attentional Bias Modification Training

Reaction times to pictures of positive parts of one’s own body before and after the ABMT for the three groups are reported in Table 2. A significant main effect of Time was found, with a decrease in reaction times from pre- to post-training across the three groups, *F*(1, 57) = 11.25, *p* = 0.001, $\eta_{p}^{2}$ = 0.17. However, there was no significant main effect of Group, indicating no difference in reaction times in the three groups across the two time points, *F*(2, 57) = 0.69, *p* = 0.507, $\eta_{p}^{2}$ = 0.02. Furthermore, the ANOVA revealed no significant Time × Group interaction, indicating that there were no differences between the three training conditions regarding the change in reaction times from pre- to post-ABMT, *F*(2, 57) = 2.18, *p* = 0.123, $\eta_{p}^{2}$ = 0.07.

**Table 2:** Means (*M*), standard deviations (SD), and effect size (*d*) of the reaction times (in milliseconds) to pictures of positive parts of one’s own body before and after the ABMT.

Training condition	Pre-training		Post-training		Cohen’s *d*
	*M*	SD	*M*	SD	*d*
Positive condition	487.12	74.77	462.08	58.71	0.33
Negative condition	489.42	102.69	483.95	114.38	0.05
Control condition	480.41	61.89	437.86	39.87	0.69

Reaction times to pictures of negative parts of one’s own body before and after the ABMT for the three groups are reported in Table 3. Again, a significant main effect of Time was observed, with a decrease in reaction times from pre- to post-training across the three groups, *F*(1, 57) = 4.96, *p* = 0.030, $\eta_{p}^{2}$ = 0.08. There was no significant main effect of Group, indicating no difference in reaction times in the three groups across the two time points, *F*(2, 57) = 0.36, *p* = 0.697, $\eta_{p}^{2}$ = 0.01. Moreover, the Time × Group interaction did not reach statistical significance, indicating that there were no differences between the three training conditions regarding the change in reaction times from pre- to post-ABMT, *F*(2, 57) = 3.01, *p* = 0.057, $\eta_{p}^{2}$ = 0.10.

**Table 3:** Means (*M*), standard deviations (SD), and effect size (*d*) of the reaction times (in milliseconds) to pictures of negative parts of one’s own body before and after the ABMT.

Training condition	Pre-training		Post-training		Cohen’s *d*
	*M*	SD	*M*	SD	*d*
Positive condition	485.76	74.24	463.69	61.28	0.30
Negative condition	479.28	94.53	487.37	127.23	−0.09
Control condition	482.41	55.74	444.10	40.58	0.69

### Comparison of the Degree of Body Dissatisfaction Before and After the Attentional Bias Modification Training

The degree of body dissatisfaction before and after the ABMT for the three groups is reported in Table 4. There was no main effect of Time, indicating no change in the degree of body dissatisfaction from pre- to post-training across the three groups, *F*(1, 55) = 2.59, *p* = 0.114, $\eta_{p}^{2}$ = 0.05. Moreover, there was no significant main effect of Group, indicating no difference in the degree of body dissatisfaction in the three groups across the two time points, *F*(2, 55) = 1.13, *p* = 0.332, $\eta_{p}^{2}$ = 0.04. Furthermore, the ANOVA revealed no significant Time × Group interaction, indicating that there were no differences between the three training conditions regarding the change in the degree of body dissatisfaction from pre- to post-ABMT, *F*(2, 55) = 0.98, *p* = 0.381, $\eta_{p}^{2}$ = 0.03.

**Table 4:** Means (*M*), standard deviations (SD), and effect size (*d*) of the degree of body dissatisfaction before and after the ABMT.

Training condition	Pre-training		Post-training		Cohen’s *d*
	*M*	SD	*M*	SD	*d*
Positive condition	5.26	1.44	5.30	1.41	−0.02
Negative condition	4.90	1.17	4.47	1.47	0.37
Control condition	5.14	1.17	4.87	1.41	0.23

### Correlations Between Attentional Bias and Changes in Body Dissatisfaction From Pre- to Post-attentional Bias Modification Training

Attentional bias before the ABMT was not significantly correlated with the change in the degree of body dissatisfaction from pre- to post-ABMT for positively evaluated body parts (*r* = −0.42; *p* = 0.071). Moreover, no significant correlation was observable between attentional bias before the ABMT and the change in the degree of body dissatisfaction from pre- to post-ABMT for negatively evaluated body parts *(r* = −0.21; *p* = 0.369).

## Discussion

The aim of the present study was to test, using a dot-probe task, whether women display an attentional bias toward negatively valenced parts of their own body and whether this attentional bias as well as body dissatisfaction can be modified by ABMT. To our knowledge, this is the first study to use this approach by presenting pictures of the participants’ own body parts in order to examine and modify an attentional bias.

Contrary to our hypothesis, no difference emerged in participants’ reaction times toward negatively and positively valenced body parts, suggesting no attentional bias toward self-defined unattractive parts of one’s own body. This finding is consistent with the results of the eye-tracking study by Tuschen-Caffier et al. (2015), in which non-clinical women displayed a balanced attentional focus on positively and negatively valenced parts of their own body. Furthermore, these results indicating balanced attention regarding one’s own body support the assumption of the integrated cognitive-behavioral model of eating disorders (Williamson et al., 2004) that attentional biases are linked to a negative body-related self-schema. As the participants in our study displayed a generally low level of eating disorder symptoms, it might be assumed that they had a less pronounced negative body-related self-schema, thus providing an explanation for the finding that they did not show biased attention. This consideration is supported by various studies using the dot-probe task (Rieger et al., 1998; Smith and Rieger, 2009; Blechert et al., 2010; Gao et al., 2011) or an eye-tracking approach (Roefs et al., 2008; Tuschen-Caffier et al., 2015; Bauer et al., 2017), which found that the extent of attentional bias toward negative stimuli is linked to the degree of body dissatisfaction.

Moreover, our hypothesis that an attentional bias as well as the degree of body dissatisfaction can be modified by ABMT based on a dot-probe task comprising pictures of the participants’ own body parts could not be confirmed. Instead, the study demonstrated that there were no changes either in reaction times to pictures of the participants’ body parts or in the degree of body dissatisfaction from pre- to post-ABMT in the three groups, indicating that there were no intervention effects. Furthermore, there was no correlation between the extent of attentional bias before the ABMT and the magnitude of change in the degree of body dissatisfaction from pre- to post-ABMT, indicating that the ABMT did not work better for participants who showed a higher extent of biased attention. In this respect, our findings are partially in accordance with the results of Smeets et al. (2011a), who also did not find an effect of the ABMT toward positively evaluated body areas, as indicated by a lack of change in body satisfaction. However, the latter authors did detect an effect of the ABMT toward negatively valenced body parts, as body satisfaction decreased after the ABMT, a finding which could not be confirmed in our study.

In this regard, the lack of effect of the ABMT toward the positively evaluated body parts might be due to the fact that the participants in our study showed a generally low level of shape and weight concerns (for reference values, see Hilbert and Tuschen-Caffier, 2016), leading to the assumption that there was little scope for improving attentional focus and for enhancing body satisfaction after the ABMT toward positively valenced body parts. This is in accordance with the assumption of the integrated cognitive-behavioral model of eating disorders (Williamson et al., 2004) that women with eating disorder symptoms display more distinctive attentional biases than women without eating disorder symptoms, with a consequently higher scope for improvement after ABMT. In this vein, results from anxiety disorder research indicate that the impact of ABMT might depend on symptom severity. For instance, in a meta-analysis, Hakamata et al. (2010) found that the effects of ABMT on anxiety symptoms are greater in clinical than in non-clinical samples. Likewise, eating disorder research has shown that the higher the degree of eating disorder and body image disturbance symptoms, the more pronounced the changeability of body image-related measures (Tuschen-Caffier et al., 2003; Vocks et al., 2009; Kraus et al., 2015). This might explain the lack of effect of the ABMT in our study, as our participants did not show clinical symptoms.

Moreover, the different results concerning the effect of the negative condition of the ABMT in our study might lie in the fact that, in contrast to Smeets et al. (2011a), who only used the three most attractive and unattractive body parts out of a total of 12 ranked body parts, we did not exclude body pictures rated in the medium range of attractiveness. This might have led to only small differences in the emotional valences of the pictures presented in the training, which did not provide a sufficient contrast to create a disparity between the training conditions.

Additionally, in contrast to the present study, participants in the study by Smeets et al. (2011a) showed a moderate degree of body dissatisfaction. As mentioned above, a higher degree of eating disorder and body image disturbance symptoms seems to be linked to a more pronounced changeability of body image-related measures (Tuschen-Caffier et al., 2003; Vocks et al., 2009; Kraus et al., 2015) and a more distinct attentional bias, with a higher scope for change in attentional bias and body dissatisfaction after ABMT (Williamson et al., 2004). In this respect, as a heightened symptom level might be linked to an increased vulnerability, it might be assumed that the participants’ heightened level of body dissatisfaction in the study by Smeets et al. (2011a) was associated with the effect of the ABMT towards negatively evaluated body parts, as indicated by a decrease in body satisfaction after the ABMT. Nevertheless, although participants showed a moderate degree of body dissatisfaction, the initial ABMT towards positively evaluated body parts did not lead to an increase in the degree of body satisfaction.

Furthermore, it should be taken into account that we conducted a single session of ABMT comprising 500 trials. As previous studies indicated that a single session delivers smaller effects compared to multisession training (Field et al., 2009; Hakamata et al., 2010; Schmitz and Svaldi, 2017), it is possible that repetitive training sessions of a longer duration and comprising a higher number of trials might have led to a change in the attentional focus and in body satisfaction.

However, as the participants in our study showed a low level of shape and weight concerns, with presumably little scope for improving attentional focus and for increasing body satisfaction after the ABMT toward positively valenced body parts, it might be assumed that multisession training would not have changed the corresponding results. There may, though, have been sufficient scope for a decrease in body satisfaction after the ABMT towards negatively valenced body parts. As we were investigating a novel ABMT approach, we aimed to examine the effects of the ABMT towards both positively and negatively evaluated body parts.

Additionally, previous studies revealed that ABMT yields greater effects when words rather than pictures are used as target stimuli in a dot-probe task (for a review, see Smith and Rieger, 2009; Hakamata et al., 2010; Renwick et al., 2013). Therefore, it cannot be ruled out that the inclusion of pictures of body parts as target stimuli might have attenuated the effects of the ABMT. Moreover, the sample size was small, leading to insufficient statistical power to detect possibly existing small group differences. Hence, replications of this study with a larger sample size are warranted. Beyond these considerations concerning sample size, further studies should focus on examining a clinical or sub-clinical sample, as it might be expected that a heightened level of shape and weight concerns provides more scope for improving attentional focus and increasing body satisfaction (Williamson et al., 2004). Due to ethical considerations, a clinical sample should merely undergo the positive and the neutral condition of the ABMT. As the negative condition of the ABMT aimed to induce an attentional focus on negatively evaluated parts of one’s own body as well as a decrease in body satisfaction, for ethical reasons, we chose a non-clinical sample to examine this novel ABMT approach in order to inform future applications in participants with eating disorder symptoms.

Finally, it should be taken into account that within the ABMT based on the dot-probe task employed in our study, the body parts were presented discretely and were therefore not integrated into a representation of the participants’ entire body. This fragmented representation of the body might have led to a limited ecological validity, and thus to a decreased transferability of the ABMT to the participants’ attentional focus in daily life as well as to body satisfaction. In this respect, as body exposure comprises the presentation of the entire body as well as its movements during mirror exposure sessions, this method for directing the attentional focus towards positively valenced body areas might lead to higher ecological validity (Vocks et al., 2007, 2008a; Griffen et al., 2018).

Overall, the results provide hints that the ABMT based on a dot-probe task, comprising pictures of the participants’ own body parts, is not a promising approach for modifying the attentional focus and the degree of body dissatisfaction. In our study in a non-clinical sample, the degree of body satisfaction was neither increased nor decreased by means of the ABMT. As such, the results suggest that this approach is not suitable for modifying attention allocation and the degree of body satisfaction. This is also in accordance with findings from anxiety disorder research, as Carlbring et al. (2012) did not succeed in modifying attentional bias in participants with social anxiety symptoms. The present study contributes to the current debate concerning the reliability of the dot-probe task as a measure of attentional bias as well as concerning the utility of ABMT based on a dot-probe task (Clarke et al., 2014). Results of previous studies examining attentional bias towards threat-related stimuli suggested that the dot-probe task shows only limited reliability for measuring attentional bias (Schmukle, 2005; Kruijt et al., 2019). To increase the reliability of a dot-probe task-based measurement, previous studies suggested that a larger sample size as well as a comparison of clinical and non-clinical samples are required (Schmukle, 2005; Kruijt et al., 2019). Although the study by Schmukle (2005) found that the dot-probe task comprising threat-related stimuli was an unreliable measure of the attentional focus in non-clinical participants, there are no hints that these results are applicable to the field of eating disorder research and to studies on the attentional focus on body-related stimuli. Furthermore, Schmukle (2005) emphasized that the findings of low reliability of the dot-probe task are not applicable to experimental treatments aiming to induce an attentional bias. Therefore, it appeared reasonable to examine and modify the attentional focus on parts of one’s own body in a non-clinical sample using the dot-probe paradigm. Beyond these considerations concerning the measurement of biased attention by means of a dot-probe task, nowadays, more reliable and adjuvant methods for measuring attentional bias are available. In this respect, as various eye-tracking studies have provided important results concerning attention allocation in clinical and non-clinical participants (Jansen et al., 2005; Roefs et al., 2008; Tuschen-Caffier et al., 2015; Bauer et al., 2017), it might be assumed that the assessment of participants’ eye movements using an eye-tracker constitutes a suitable approach for measuring the attentional focus on one’s own body.

In conclusion, previous research indicates that body-related biased attention seems to contribute to the etiology and maintenance of body dissatisfaction, which is postulated to constitute a risk factor in the development of eating disorders. However, as eating disorder symptoms appear to persist despite psychotherapeutic treatments, it is of particular importance to generate effective interventional methods addressing body-related attentional biases. In this regard, further examinations are needed and should take the considerations and conclusions of this study into account.

## Data Availability Statement

The datasets for this manuscript are not publicly available because the local ethics committee of Osnabrück University stipulated that data must not be passed on to third parties. Request to access the datasets should be directed to the corresponding author.

## Ethics Statement

The studies involving human participants were reviewed and approved by the Ethics committee of the Osnabrück University. The patients/participants provided their written informed consent to participate in this study.

## Author Contributions

SV, MW, and AV-E planned and conducted the study. AV-E programmed the experimental procedure and was involved in the development of the stimuli manipulation procedure. NE, SV, MW, and AH analyzed the data. NE wrote the first draft of the manuscript. All authors contributed to the compilation of the manuscript and read and approved the submitted version.

### Conflict of Interest

The authors declare that the research was conducted in the absence of any commercial or financial relationships that could be construed as a potential conflict of interest.
